# Ultra-lightweight tomatoes disease recognition method based on efficient attention mechanism in complex environment

**DOI:** 10.3389/fpls.2024.1491593

**Published:** 2025-02-13

**Authors:** Wenbin Sun, Zhilong Xu, Kang Xu, Lin Ru, Ranbing Yang, Rong Wang, Jiejie Xing

**Affiliations:** ^1^ College of Information and Communication Engineering, Hainan University, Haikou, China; ^2^ College of Mechanical and Electrical Engineering, Hainan University, Haikou, China; ^3^ College of Civil Engineering and Water Conservancy, Heilongjiang Bayi Agricultural University, Daqing, China; ^4^ Information Technology Research Center, Beijing Academy of Agriculture and Forestry Sciences, Beijing, China

**Keywords:** plant disease identification, image classification, attention mechanism, deep separable convolution, deep learning

## Abstract

A variety of diseased leaves and background noise types are present in images of diseased tomatoes captured in real-world environments. However, existing tomato leaf disease recognition models are limited to recognizing only a single leaf, rendering them unsuitable for practical applications in real-world scenarios. Additionally, these models consume significant hardware resources, making their implementation challenging for agricultural production and promotion. To address these issues, this study proposes a framework that integrates tomato leaf detection with leaf disease recognition. This framework includes a leaf detection model designed for diverse and complex environments, along with an ultra-lightweight model for recognizing tomato leaf diseases. To minimize hardware resource consumption, we developed five inverted residual modules coupled with an efficient attention mechanism, resulting in an ultra-lightweight recognition model that effectively balances model complexity and accuracy. The proposed network was trained on a dataset collected from real environments, and 14 contrasting experiments were conducted under varying noise conditions. The results indicate that the accuracy of the ultra-lightweight tomato disease recognition model, which utilizes the efficient attention mechanism, is 97.84%, with only 0.418 million parameters. Compared to traditional image recognition models, the model presented in this study not only achieves enhanced recognition accuracy across 14 noisy environments but also significantly reduces the number of required model parameters, thereby overcoming the limitation of existing models that can only recognize single disease images.

## 1 Introduction

Tomatoes, as a widely cultivated and significant crop, possess considerable edible and medicinal value. The diagnosis of early diseases typically relies on the assessment of leaf damage by experts. However, the inability of these experts to provide real-time diagnoses often results in missed opportunities for timely prevention, which can lead to substantial economic losses. Consequently, the development of an automatic and efficient crop disease diagnosis system has become an urgent area of research.

In early research, scholars aimed to leverage computer vision technology for the automatic identification of diseases. Gulhane and Gurjar (2011) employed a Back Propagation Neural Network (BPNN) to identify cotton diseases by extracting relevant disease characteristics. Sannakki et al. (2010) utilized a fuzzy algorithm in conjunction with K-means clustering (Bashish et al., 2011) to facilitate the grading of leaf diseases. Xie et al. (2016) developed a diagnostic system for wheat leaf diseases based on an Android smartphone, which effectively reduced the computational complexity associated with automated algorithms. Additionally, Siricharoen et al. (2016) diagnosed plant diseases by analyzing leaf textures, colors, and shapes. Qin et al. (2016) employed K-means clustering, the Naive Bayes algorithm, regression trees, and various other supervised classification methods to identify alfalfa diseases, ultimately seeking the optimal method for disease identification in alfalfa. However, the reliance on manually designed features necessitates expert knowledge, which not only limits the degree of automation but also renders the system less adaptable to accommodate variations in growth conditions such as light intensity and complex backgrounds when only a few related features are utilized. Consequently, both recognition accuracy and the level of automation in complex environments require further enhancement.

With the development of computer vision technology and internet technology (Keswani et al., 2019, 2020), the application of deep learning algorithms (Krizhevsky et al., 2017; Simonyan and Zisserman, 2014; He et al., 2016) to crop disease recognition has shown great potential (Dyrmann et al., 2016; Mohanty et al., 2016). Jiang et al. (2020) used deep learning methods to extract the disease characteristics of tomato leaves, such as spot blight, late blight, and yellow leaf curl. Their method predicted the category of each disease after continuous iterative learning, and the accuracy achieved on the training dataset and testing dataset were increased by 0.6% and 2.3%, respectively. Lv et al. (2020) proposed a framework to identify corn leaf diseases based on an improved AlexNet network and feature enhancement, thus improving the extraction performance of corn disease features in complex environments and increasing the accuracy of disease recognition. Liu et al. (2020) used DenseNet to train a generative adversarial network to generate images of 4 different leaf diseases and proposed a leaf disease recognition model based this network. It was further verified that data enhancement could not only effectively overcome the overfitting problem in disease recognition but also effectively improve the recognition accuracy. Liang and Zhang (2020) integrated three classifiers for plant disease image recognition, and the accuracy of the proposed method on a segmentation testing dataset was close to 99.92%. Huang et al. (2019) proposed an end-to-end plant disease diagnosis model based on deep neural networks that could reliably identify plant types and diseases. Sanida and Dasygenis (2024) proposed a lightweight convolutional neural network for tomato leaf disease identification, achieving an accuracy of 97.63% and an AUC score of 98.51%. Ni et al. (2023) introduced a tomato leaf disease recognition model based on ResNet18, enhanced by the addition of a squeeze-and-excitation module, which attained an average recognition accuracy of 99.63% on the publicly available PlantVillage dataset. Li et al. (2023) developed a tomato leaf disease identification model utilizing a self-attention mechanism, achieving an accuracy of 99.97% with a parameter size of 27 MB on the PlantVillage dataset. Additionally, Yang et al. (2024) proposed a lightweight CNN model, which demonstrated a recognition accuracy of 95.54%.These studies have proven that data enhancement can improve the robustness of models in different environments, but such recognition methods still have some shortcomings. (1) These methods all improve the recognition accuracy of the developed models by sacrificing complexity, lacking focus on the model’s complexity. (2) The input images of these end-to-end models can contain only one leaf. If the leaf size is small, the background is large, or multiple diseased leaves appear in one image, the models cannot recognize it. (3) Robustness studies regarding the data variability caused by image acquisition devices are scarce.

Therefore, to overcome these problems, this paper presents a framework that combines tomato leaf detection and leaf disease identification and is mainly divided into three parts. First, a tomato leaf detection model is designed to detect and crop tomato leaves in captured images to solve the problem of non-singular disease image recognition. Then, an effective data enhancement method is designed to improve the robustness of the model in complex environments. Finally, an ultra-lightweight tomato disease recognition model is designed to reduce required the number of model parameters while ensuring that the recognition accuracy remains unchanged and to balance the contradictory relationship between the complexity and recognition accuracy of the model.

## 2 Related work

To build a tomato leaf detection model in a complex environment, it is necessary to select a suitable object detection network. Mainstream object detection algorithms based on deep learning are mainly divided into two categories, and different types of object detection algorithms have different performances. The first category contains single-step detection networks that do not generate proposal regions but can directly convert an object frame positioning problem into a regression processing problem to achieve rapid object detection; such methods include You Only Look Once (YOLO) (Redmon et al., 2016), YOLOv3 (Redmon & Farhadi, 2018), YOLOv4 (Bochkovskiy et al., 2020), YOLOv6 (Li et al., 2022), YOLOv7 (Wang et al., 2022), YOLOv9 (Wang et al., 2024b), YOLOv10 (Wang et al., 2024a) and other networks, but their disadvantage is that they are prone to missing and falsely detected objects. The second type includes two-step detection networks based on proposal regions, which have high detection accuracy and positioning accuracy, and the probabilities of missed detections and false detections are relatively small; such methods include the fast region-convolutional neural network (Fast R-CNN) (Girshick, 2015) (Fast Region-Convolutional Neural Network) and Faster R-CNN (Ren et al., 2017). The tomato leaf detection results directly affect the accuracy of the utilized disease recognition model, so how to accurately detect tomato leaves in a complex background environment is particularly important. In this paper, we choose Faster RCNN as the basis for constructing a tomato leaf detection network due to its higher accuracy.

The recognition accuracy of image classification algorithms based on deep learning is superior to that traditional algorithms. VGG (Simonyan and Zisserman, 2014), ResNet (He et al., 2016), and GoogLeNet (Szegedy et al., 2015) realize deep convolutional neural networks (DCNNs) by increasing the number of utilized convolutional layers and widening their network structures, resulting in stronger feature extraction capabilities but requiring many hardware resources. To reduce the number of model parameters, MobileNetV1 (Howard et al., 2017) uses deep separable convolution to significantly improve computational efficiency, while MobileNetV2 (Sandler et al., 2018) employs a resource-efficient block with an inverse residual and a linear bottleneck to extend it. MobileNetV3 (Howard et al., 2019) introduces an attention mechanism and modifies the number of extended layer filters. These MobileNet series networks have become mainstream lightweight models, as they greatly reduce the number of required model parameters under the premise of ensuring recognition accuracy. Although MobileNetV3 incorporates an attention mechanism into the lightweight network and achieves better recognition performance, the introduced attention mechanism uses two fully connected layers to perform dimensionality reduction and then upgrade operations on channel features, resulting in an increased number of parameters and greater feature losses. Therefore, this paper designs an ultra-lightweight tomato disease recognition model based on the MobileNet series of models.

Because the image features of early diseased leaves are not obvious and a large number of network parameters can be used to extract the texture features of leaves, few studies have been conducted on the use of lightweight models for crop disease identification. If a lightweight network is used for disease image recognition, it is necessary to improve the feature extraction ability of the model and the utilization of model parameters to extract the features of diseased leaves at different scales. An attention mechanism enables a network to pay more attention to the most effective information in the image and ignore the irrelevant information, so it is considered an effective module for the aggregation of enhanced features. The squeeze-and-excitation network (SE-Net) (Jie et al., 2017) first proposed network that included an effective mechanism for learning channel attention, and it has achieved good performance. A convolutional block attention module (CBAM) (Woo et al., 2018) uses both average pooling and maximum pooling to aggregate features. Most of the attention modules proposed later, such as the Gram-Schmidt orthogonalization procedure (GSoP) (Gao et al., 2018) and Gather-excite (GE) (Hu et al., 2018), have high model complexity and can only be used in a single block or several convolutional blocks. Notable, all the above methods focus on developing complex attention modules to achieve better performance. An efficient attention mechanism aims to learn effective channel attention with low model complexity. The introduction of a high-efficiency attention mechanism into a tomato disease recognition model can reduce the impact of model weight on the resulting recognition accuracy.

Therefore, this paper defines a framework that combines tomato leaf detection and tomato disease recognition, divides tomato the disease recognition into three parts, and optimizes the method used for each part according to tomato disease characteristics to make it suitable for tomato disease recognition in real scenes. The main contributions of this article are as follows:

1. By using MobileNetV2 to improve the feature extraction module of Faster RCNN, a fast detection method for tomato leaves in a complex environment is proposed to cut out the complete leaves in the camera’s field of view from the background.
2. The existing tomato disease dataset was formed by picking diseased leaves and taking their pictures in an ideal experimental environment, so it lacks disease images in real growth environments and complex environments. Therefore, this paper collects tomato leaf images in an unconstrained tomato planting environment and designs a set of data enhancement methods to enhance the robustness of the recognition model in complex environments.
3. An ultra-lightweight tomato disease recognition model based on an efficient attention mechanism is proposed; it balances the contradictory relationship between model complexity and accuracy.

## 3 Materials and methods

**Figure 1:**
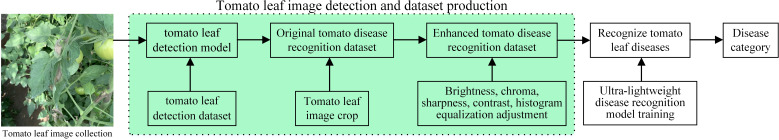
A framework for the fusion of tomato leaf detection and leaf disease recognition.

Data collection is an important part of crop disease identification. In this paper, tomato leaf images were collected at the tomato planting base of the Beijing Agricultural Information Technology Research Center. Under natural light, a Sony camera was used to capture tomato images with resolutions of 3024×4023 and 3024×3024. The disease image collection times were 6:00-8:00 am, 10:00-12:00 am and 2:00-6:00 pm. The collected disease category database includes powdery mildew, leaf mould and late blight, with a total of 2437 images. The images collected in an unconstrained environment are stored according to their disease categories. It is worth noting that to increase the diversity of the dataset, the photographed tomato disease images not only include tomato leaves, stems and fruits but also images collected at different angles, at different scales and with different background information for the same leaf. These complex environments, with characteristics such as light, dirt, healthy stems and leaves, the ground, human hands, and other objects, affect the results of tomato disease recognition. Therefore, this paper designs a framework that integrates tomato leaf detection and leaf disease recognition and proposes a rapid leaf detection model and an ultra-lightweight tomato disease recognition model that is suitable for complex environments. As shown in **Figure 1**, the framework is mainly divided into two parts. The first part mainly uses an object detection model to obtain a dataset of tomato leaf diseases, and then enhances the data of tomato leaves. A lightweight leaf recognition model is constructed using the enhanced tomato leaf disease data, and the trained recognition model is used to recognize leaf diseases.

### 3.1 Tomato leaf detection in a complex environment

#### 3.1.1 Data preparation

Pascal VOC2007 (Ren et al., 2017) is a public dataset in the field of object detection. To train the tomato leaf detection model, 800 images are extracted from among the collected images as the tomato leaf detection dataset and annotated according to the Pascal VOC2007 format. According to the standard that the target area accounts for more than 3/4 of the entire leaf area, it is considered a complete leaves. ImgLabel software is used to manually select complete healthy leaves and diseased leaves with clear pixels as the target areas, and fuzzy, incomplete leaves and other background information are uniformly defined as the image background and label labelled. The annotated images generate labels in Extensible Markup Language (XML) format as the tomato leaf detection dataset. (Xu et al., 2024), the dataset are randomly divided in an 8:1:1 ratio, resulting in 648 training images, 80 testing images, and 72 validation images.

#### 3.1.2 Tomato leaf detection model

**Figure 2:**
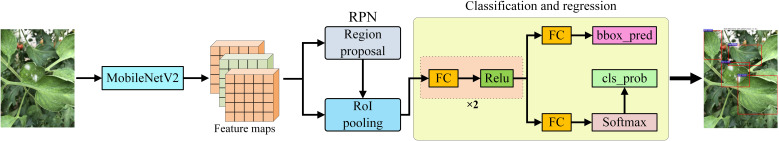
Structure of the tomato leaf detection model.

The accuracy of tomato leaf detection is directly related to the accuracy of the disease identification model. Currently, mainstream target detection models are mainly divided into two categories: single-stage and two-stage. Two-stage target detection algorithms consist of two steps: first, generating candidate regions, and then applying a classifier to these regions. This method is more accurate than single-stage detection but slower. Considering that the detection effect of leaves significantly impacts subsequent results, this paper constructs a tomato leaf detection model based on the Faster RCNN, which has high accuracy in two-stage target detection networks, as shown in **Figure 2**.

**Figure 3:**
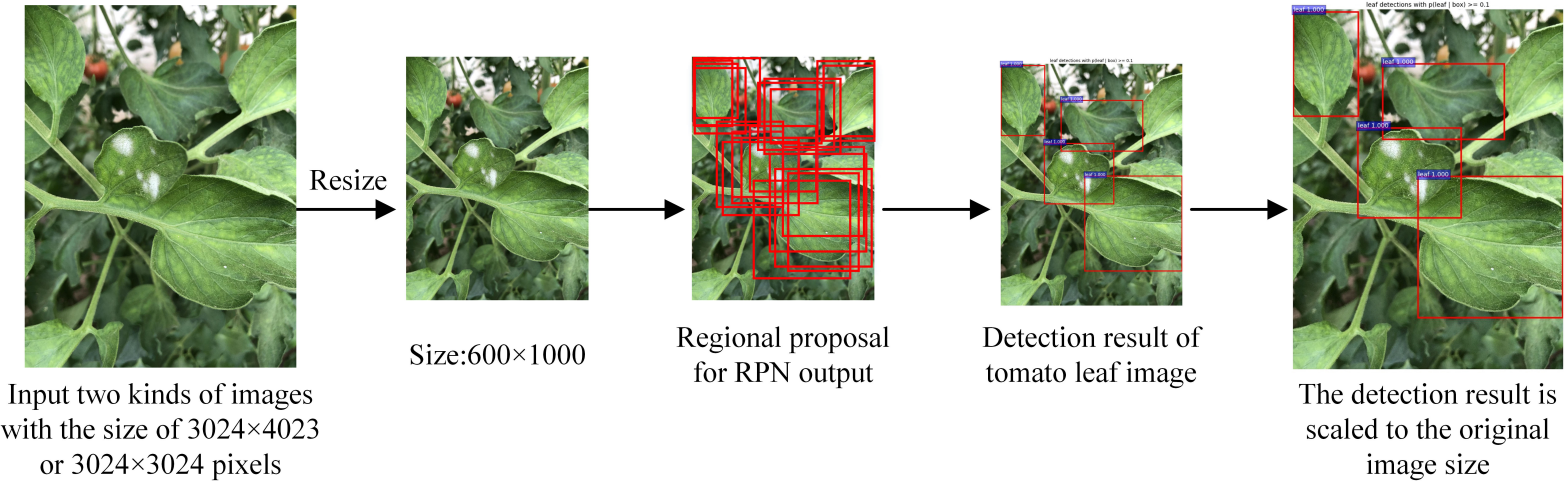
Schematic diagram of the inference process of the tomato leaf detection model.

The tomato leaf detection model includes a feature extraction network, region proposal network (RPN) and leaf classification and regression network. MobileNetV2 is used as the feature extraction network in Faster RCNN to extract tomato leaf features, reduce the number of model parameters, and then input the generated feature map into the RPN to search for a predefined number of suggested regions. The feature map output by MobileNetV2 and the region proposal output by the RPN are input into the region of interest (RoI) pooling layer, and after being adjusted to a fixed size, each leaf area is input into the leaf classification and regression network to detect and identify tomato leaves. The reasoning process of the tomato leaf detection model is shown in **Figure 3**.

Each image is scaled to a size of 600×1000 pixels and input into the tomato leaf detection model to detect all clear and complete tomato leaf coordinates in the image, and the output detection results are rescaled to fit the original image. When there are no leaves in the given image, the number of detected leaves is 0, and the image is not saved. The tomato leaf detection model accurately extracts all clear and complete leaf images in the camera’s field of view, thereby overcoming the limitation of current disease recognition research that the input image can only contain one leaf. This paper automatically detects images containing multiple tomato leaves and inputs the detected single leaves into the disease recognition network, which not only improves the accuracy and adaptability of tomato disease detection but also greatly reduces the workloads of researchers.

#### 3.1.3 Tomato disease image enhancement

**Figure 4:**
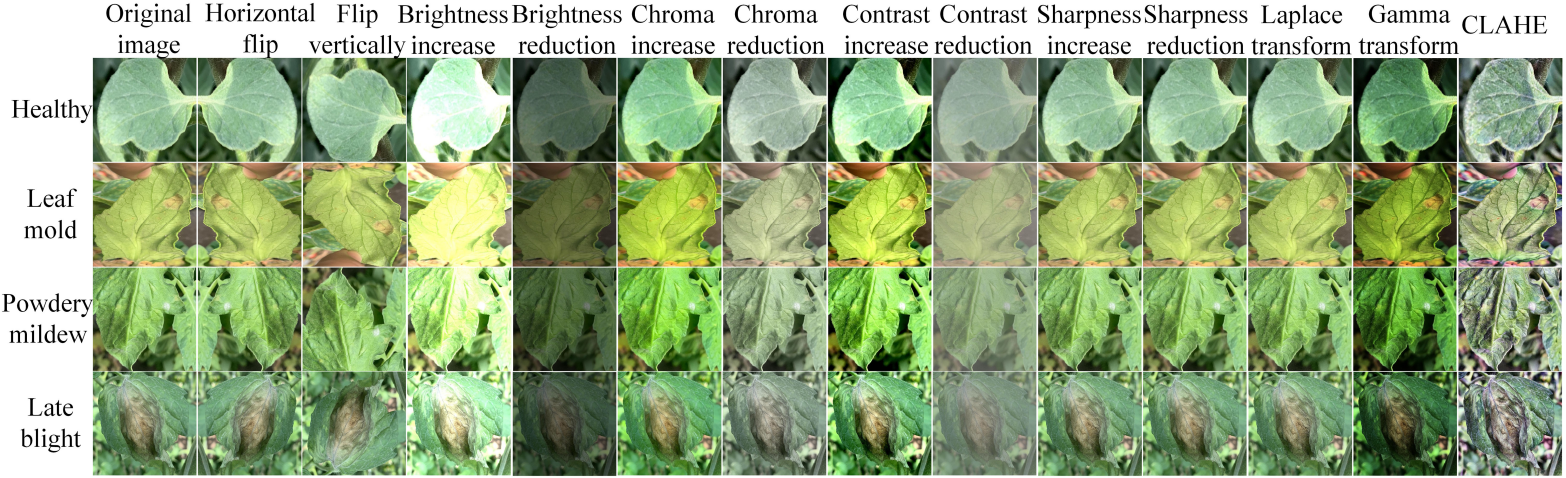
The results of tomato disease image enhancement.

After the complete tomato leaves are cut out from the images taken by the camera, they are divided into four categories: powdery mildew, leaf mould, late blight and healthy leaves. These four kinds of disease data form the original tomato disease recognition dataset, which is divided into a training set and a test set at a ratio of 8:2. To enhance the robustness of the tomato disease recognition model in an unconstrained environment, this paper uses 13 data enhancement methods to expand the number of training images and test images. The enhancement results are shown in **Figure 4**.

Image enhancement methods include flipping an image horizontally or vertically and increasing or decreasing the brightness, chroma, contrast, or sharpness of the image. These methods can simulate special lighting noise in a real environment, not only increasing the diversity of the training set but also enabling the design of comparative experiments to test the robustness of the developed model in a complex and changeable environment. In the image field, the Laplacian algorithm can highlight the edge information of an image, the gamma transform can perform different contrast enhancements according to different grey values, and contrast-limited adaptive histogram equalization (CLAHE) can enhance the local contrast of an image and increase the number of detailed features. The tomato disease dataset expanded using these methods is called the enhanced tomato disease recognition dataset, in which the brightness, chroma, contrast, etc. are increased to 50% of the original image or reduced to 50% of those of the original image.

### 3.2 Ultra-lightweight tomato leaf disease recognition model

**Figure 5:**
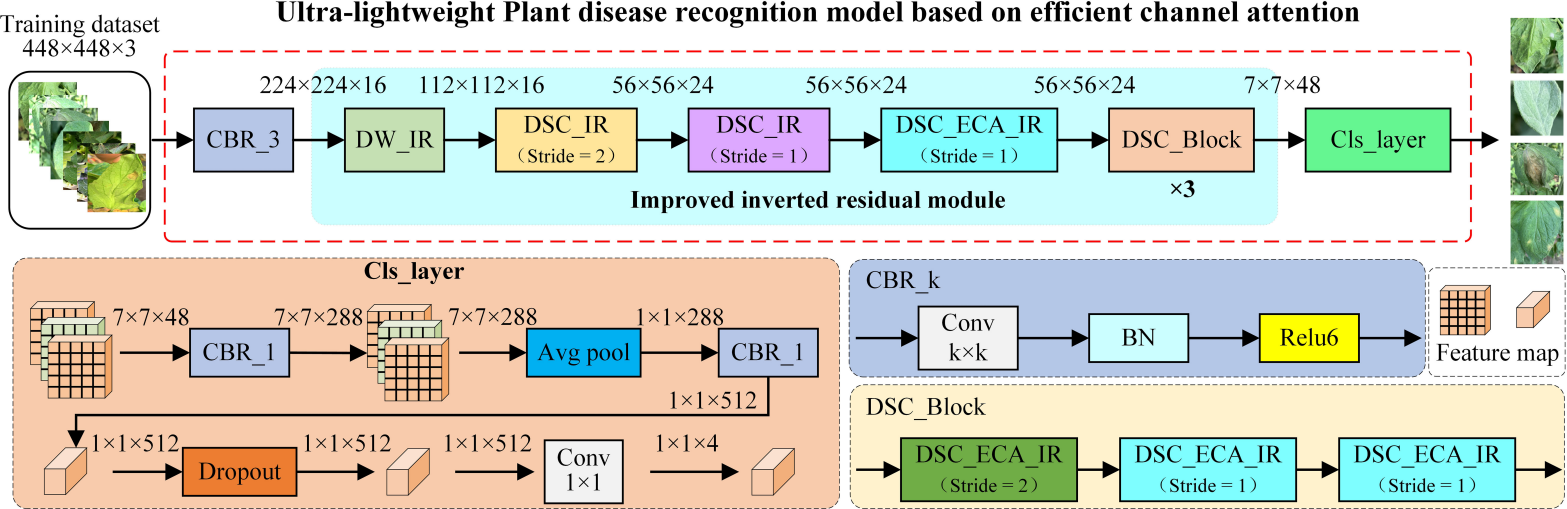
Structure of an ultra-lightweight Plant leaf recognition model based on ECA.

At present, the mainstream recognition model has a relatively high number of parameters and consumes a relatively large amount of hardware resources. To balance the contradictory relationship between accuracy and complexity and to facilitate the deployment and application of the model, this paper uses deep separable convolution, an inverted residual structure and efficient channel attention (ECA) to design an ultra-lightweight tomato leaf disease recognition model. The specific architecture is shown in **Figure 5**.

To preserve more detailed diseased leaf features, five improved inverted residual structures are designed in the model, based on MobileNet v3 small, and the input image size of the model is set to 448×448 size. After a tomato leaf image feature is extracted by the case-based reasoning (CBR) module, the obtained feature map is input into the feature extraction network, which contains 5 improved inverted residual modules. Finally, the designed last-stage module uses a 1×1 convolution instead of a fully connected layer and uses a global pooling layer to transform 7×7 feature maps into 1×1 objects, which greatly reduces the number of required parameters. A dropout layer is added between the last two 1×1 convolutional layers of the model to prevent the model from overfitting. The parameters of each module are shown in **Table 1**.

**Table 1:** Specific parameters of each module.

Input	Module	Expansion channel	Output channel	ECA	Step	Output size
448×448×3	Conv2d, 3×3	—	16		2	224×224×16
224×224×16	DW_IR, 3×3	16	16	√	2	112×112×16
112×112×16	DSC_IR, 3×3	72	24		2	56×56×24
56×56×24	DSC_IR, 3×3	88	24		1	56×56×24
56×56×24	DSC_ECA_IR, 3×3	88	24	√	1	56×56×24
56×56×24	DSC_ECA_IR, 5×5	96	40	√	2	28×28×40
28×28×40	DSC_ECA_IR, 5×5	240	40	√	1	28×28×40
28×28×40	DSC_ECA_IR, 5×5	240	40	√	1	28×28×40
28×28×40	DSC_ECA_IR, 5×5	120	48	√	2	14×14×48
14×14×48	DSC_ECA_IR, 5×5	144	48	√	1	14×14×48
14×14×48	DSC_ECA_IR, 5×5	288	48	√	1	14×14×48
14×14×48	DSC_ECA_IR, 5×5	288	48	√	2	7×7×48
7×7×48	DSC_ECA_IR, 5×5	288	48	√	1	7×7×48
7×7×48	DSC_ECA_IR, 5×5	288	48	√	1	7×7×48
7×7×48	Conv2d, 1×1	–	288	–	1	7×7×288
7×7×288	Avgpool, 7×7	–	–	–	1	1×1×288
1×1×288	Conv2d, 1×1	–	512	–	1	1×1×512
1×1×512	Dropout	–	–	–	–	1×1×512
1×1×512	Conv2d 1×1	–	4	–	1	1×1×4

The pointwise convolution channel in each module is called the expansion channel of the module.

The '√' indicates whether the ECA module is used.

The model uses rectified linear unit 6 (Relu6) as the non-linear activation function and limits the maximum value of the output to 6. The calculation method is shown in equation (1):


(1) Relu6x=min(max(0,x),6)

**Figure 6:**
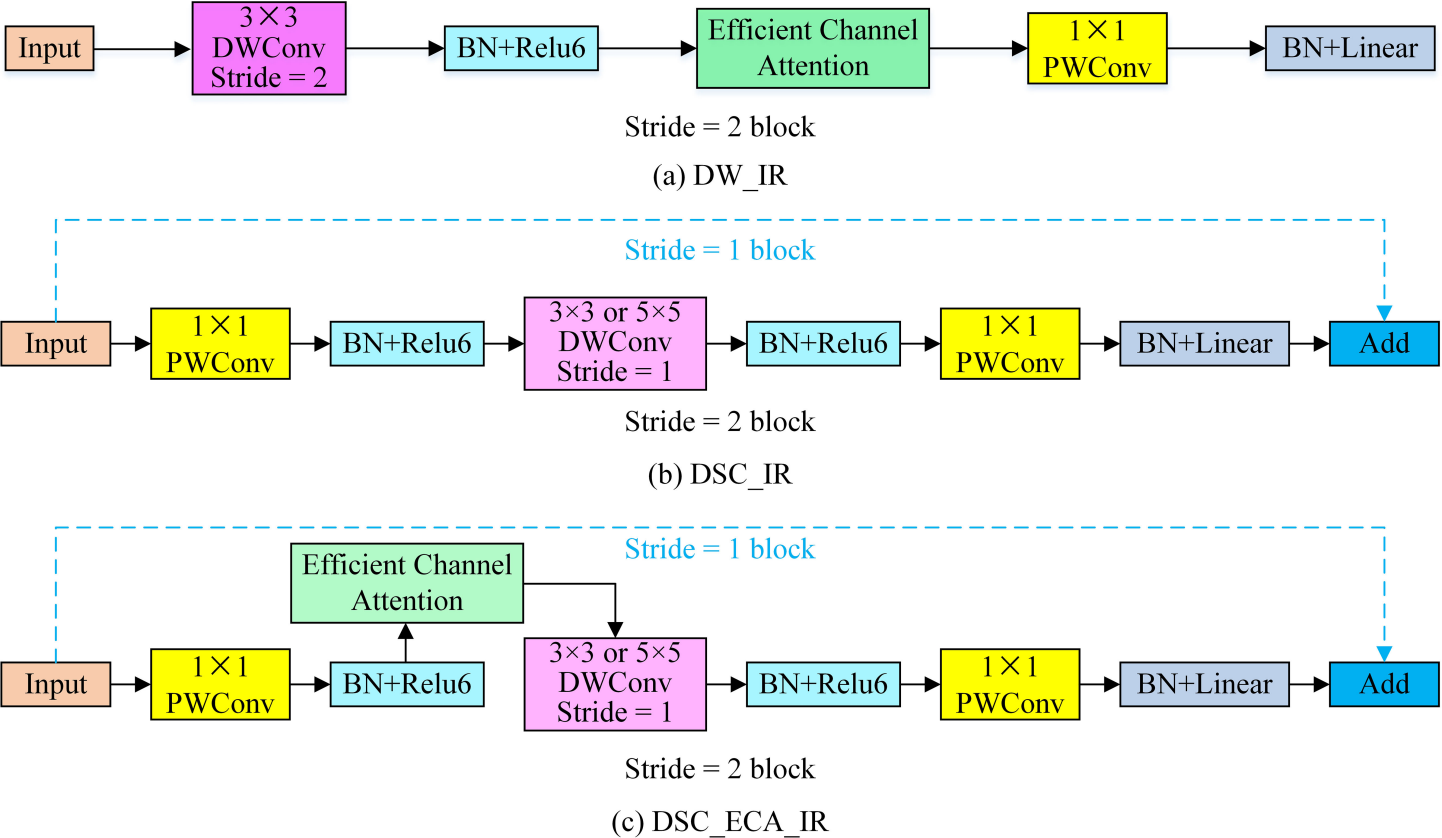
The improved inverted residual module. **(A)** DW_IR is an inverted residual module based on depth-wise convolution and an efficient attention mechanism. **(B)** is an inverted residual module based on depth-wise separable convolution (DSC_IR). **(C)** is an inverted residual module based on depth-wise separable convolution and an efficient attention mechanism (DSC_ECA_IR).

The deep network easily causes gradient degradation when extracting tomato disease characteristics. The traditional residual structure performs compression first and then conducts expansion, thus losing much of the effective information contained in the feature map. Therefore, this paper designs five inverted residual structures to perform feature extraction after upgrading the channel information, and the structures are shown in **Figure 6**.

To reduce the number of parameters required by the tomato disease recognition model, depth-wise separable convolution (DSC), which is composed of depth-wise (DW) convolution and point-wise (PW) convolution, is introduced. The use of DSC to replace the traditional standard convolution can reduce the numbers of model parameters and calculations. The inverted residual (IR) module uses PW convolution to upscale the channels and then uses DW convolution to extract features so that the model can extract rich feature information. Finally, PW convolution is used to keep the dimensionality of the input channel consistent with that of the output channel, thus achieving cross-layer connections and lightweight models. Since tomato leaf disease is mainly manifested in the texture information of tomatoes, to enhance the ability of the model to extract detailed leaf image features, an ECA mechanism is incorporated into the inverted residual structure. The module in **Figure 6A** does not perform dimensional upscaling and directly inputs the given feature map into the DW convolution mechanism. The IR modules in **Figure 6B** and **Figure 6C** both use PW convolution, DW convolution, and more PW convolution operations to increase the dimensionality of the input features and then extract the features. When the DW convolution step length is 1, a cross-layer connection is adopted, and when the step length is 2, a cross-layer connection is not adopted. In the last PW convolution, only a batch normalization (BN) structure is used, and the non-linear activation function is not used.

The standard convolution input is a feature map *L_i_* of size $h_{i}\times w_{i}\times d_{i}$ , an output feature map *L_j_* of size $h_{i}\times w_{i}\times d_{j}$ is generated by the convolution kernel $K\in R^{k\times k\times d_{i}\times d_{j}}$ , and the calculation cost is $h_{i}\cdot w_{i}\cdot d_{i}\cdot d_{j}\cdot k\cdot k$ . The DSC mechanism uses two layers instead of the standard convolution operation. The first layer is a DW convolution layer, which performs lightweight filtering on each input channel, and the second layer is a 1×1 point-by-point convolution layer, which constructs new features by calculating linear combinations of the input channels. However, the effect of DSC is similar to that of standard convolution, but the computational cost, which is the sum of a DW convolution and a 1×1 PW convolution, is lower:


(2) $$h_{i}\cdot w_{i}\cdot d_{i}(k^{2}+d_{j})$$

where the DW convolution in the lightweight model designed in this paper uses 3×3 and 5×5 convolution operations, and the computational cost is reduced by 9-25 times compared with that of traditional convolution.

**Figure 7:**
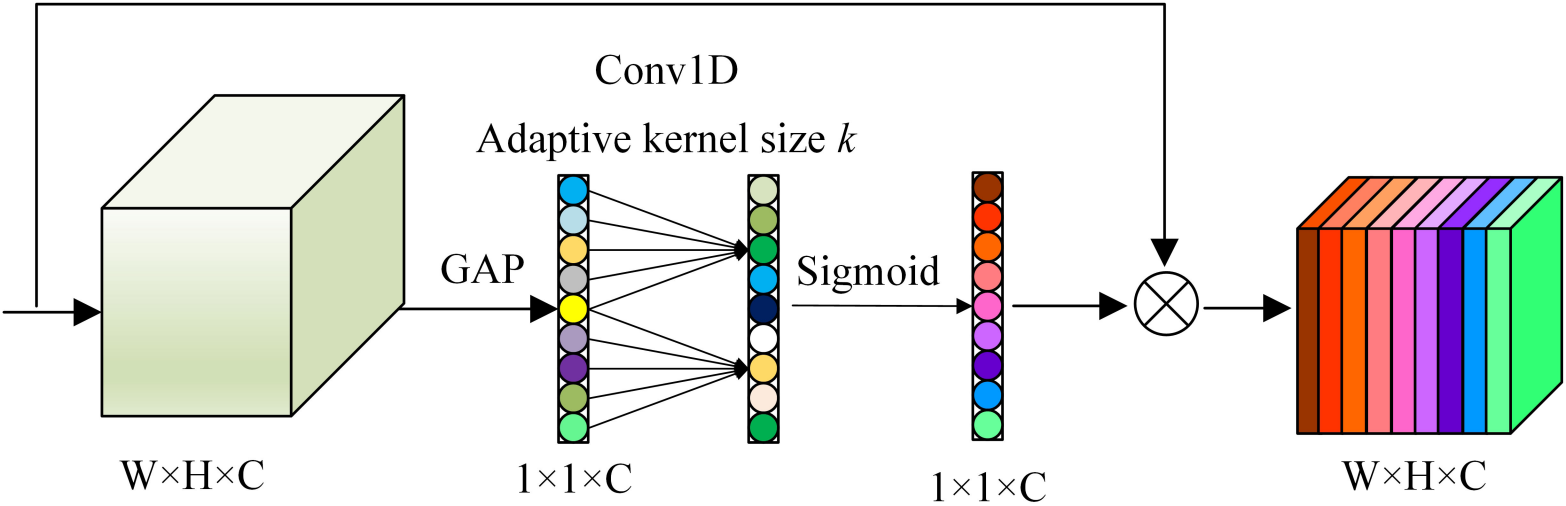
The specific structure of the ECA mechanism.

Reducing the number of model parameters can easily reduce the resulting recognition accuracy. To improve the accuracy of tomato disease recognition, this paper introduces an ECA mechanism to improve the inverted residual modules, as shown in **Figures 6A, C**. The specific structure of the ECA mechanism is shown in **Figure 7**.

A feature map X is output after a convolution-based transformation as $u_{c}(i,j)\in R^{W\times H\times C}$ , where W, H and C are the width, height and channel dimensions, respectively. The attention module uses a compression operation to compress a feature map U into a 1×1×C format. This operation aggregates features across the spatial dimensions (H×W), generates channel descriptors, and obtains aggregate features $F(u_{c})\in R^{C}$ without dimensionality reduction. The channel attention can be learned by formula (3).

The channel attention can be learned by formula (3).


(3) $$\omega =\sigma (MF(u_{c}))$$

where σ is a sigmoid function, which is the activation function of the attention module, *M* is the convolution calculation:


(4) $$sigmoid(z)=\frac{1}{1+e^{-z}}$$

The ECA module uses global average pooling (GAP) to obtain an aggregate feature $F_{A}(u_{c})$ :


(5) $$F_{A}(u_{c})=\frac{1}{H\times W}\sum_{i=1}^{H}\sum_{j=1}^{W}u_{c}(i,j)$$

When the convolution operation extracts features, the variance of the estimated value is easily increased due to the limited size of the neighborhood. GAP can reduce this error and retain more image background information.

To capture local cross-channel interactions and ensure the effectiveness of channel features, we can use a 1D convolution operation to make all channels share the same learning weight. The calculation of $\omega_{i}$ is dependent on $F_{i}(u_{c})$ and its k neighbors, i.e.,


(6) $$\left\{ \begin{array}{c} \omega_{i}=\sigma (\sum_{j=1}^{k}\omega^{j}F_{i}^{j}(u_{c})),\;F_{i}^{j}(u_{c})\in \varphi_{i}^{k}\\ \omega =\sigma (Conv1D_{k}(F_{i}(u_{c})))\;\;\;\;\;\;\;\;\;\;\;\;\;\;\;\;\;\;\;\; \end{array}\right.$$

where $\varphi_{i}^{k}$ indicates the set of *k* adjacent channels of $F_{i}^{j}(u_{c})$ . $Conv1D_{k}$ is a 1*D* convolution with a convolution kernel of k, and formula (6) involves only k parameters. Under the premise of low complexity, the efficiency and effectiveness of the model are ensured by appropriately capturing local cross-channel interactions.

Therefore, the *M* operation in formula (3) is a one-dimensional convolution operation, and the aggregate feature $F_{A}(u_{c})$ is input into the 1*D* convolution layer to filter the effective channel information and obtain $\omega_{C}$ :


(7) $$\omega_{c}=\;\sigma (Conv1D_{k}(F_{A}(u_{c})))$$

The convolution kernel size *k* is set as an adaptive parameter. For a given number of channels *C*, *k* is calculated as follows:


(8) $$k=\tau (C)=|\frac{\log_{2}(C)}{\gamma }+\frac{b}{\gamma }{|}_{odd}$$

where γ=2,  b=1, $|\alpha {|}_{odd}$ represents the nearest odd number of α and τ(C) is a nonlinear mapping.


(9) $$x_{c}=F_{scale}(u_{c},\omega_{C})=\omega_{C}\cdot u_{c}$$

where $X=[x_{1},x_{2},\ldots ,x_{c}]$ and $F_{scale}(u_{c},\omega_{C})$ represent the channel multiplication relationship between the scalar $\omega_{C}\;$ and the feature map. The ECA module does not reduce the dimensionality of the original channel features. High-dimensional channels have longer-range interactions, which are not limited to the local acceptance domain of the convolution response, and realize the enhancement and recalibration of important features in the former and latter layers.

## 4 Experimental results and analysis

### 4.1 Experimental configuration and data

The models are trained on one NVIDIA Tesla P100 GPU with 16 GB of RAM based on a 64-bit Ubuntu 16.04 operating system and the PyTorch framework, with Python version 3.7.6, PyTorch version 1.3.0, CUDA API version 10.0, and cuDNN version 7.5.1.

According to the data preprocessing procedure in **Figure 1**, the original tomato disease recognition dataset and the enhanced tomato disease recognition dataset are produced, as shown in **Table 2**. The original collected tomato disease dataset contains 3 kinds of diseases, and a total of 2,437 tomato images with two sizes, 3024×4023 and 3024×3024. To train the tomato leaf detection model, 800 images are extracted from the collected images and manually labelled as the tomato leaf detection dataset. Finally, the overall dataset is divided into 80 testing images, 648 training images and 72 validation images. The trained tomato leaf detection model is used to crop the tomato leaves with powdery mildew, late blight, leaf mould and healthy tissues from the original image, and 6,001 leaf images are obtained; these form the original tomato disease recognition dataset and are divided into a training set and testing set at a ratio of 8:2. Thirteen kinds of image enhancement methods are used to expand the training set and testing set of the original tomato disease recognition, and 84,014 enhanced leaf images are obtained, forming the enhanced tomato disease recognition dataset.

**Table 2:** Number of images in the tomato disease dataset.

Category	Captured image	Tomato leaf image crop	Original tomato disease recognition dataset		Image enhancement	Enhanced tomato disease recognition dataset	
			Training set	Testing set		Training set	Testing set
Powdery mildew	460	968	775	193	13552	10850	2702
Late blight	1231	2335	1868	467	32690	26152	6538
Leaf mould	746	1441	1153	288	20174	16142	4032
Healthy	0	1257	1006	251	17598	14084	3514
Total	2437	6001	4802	1199	84014	67228	16786

### 4.2 Testing results of the tomato leaf detection model

**Figure 8:**
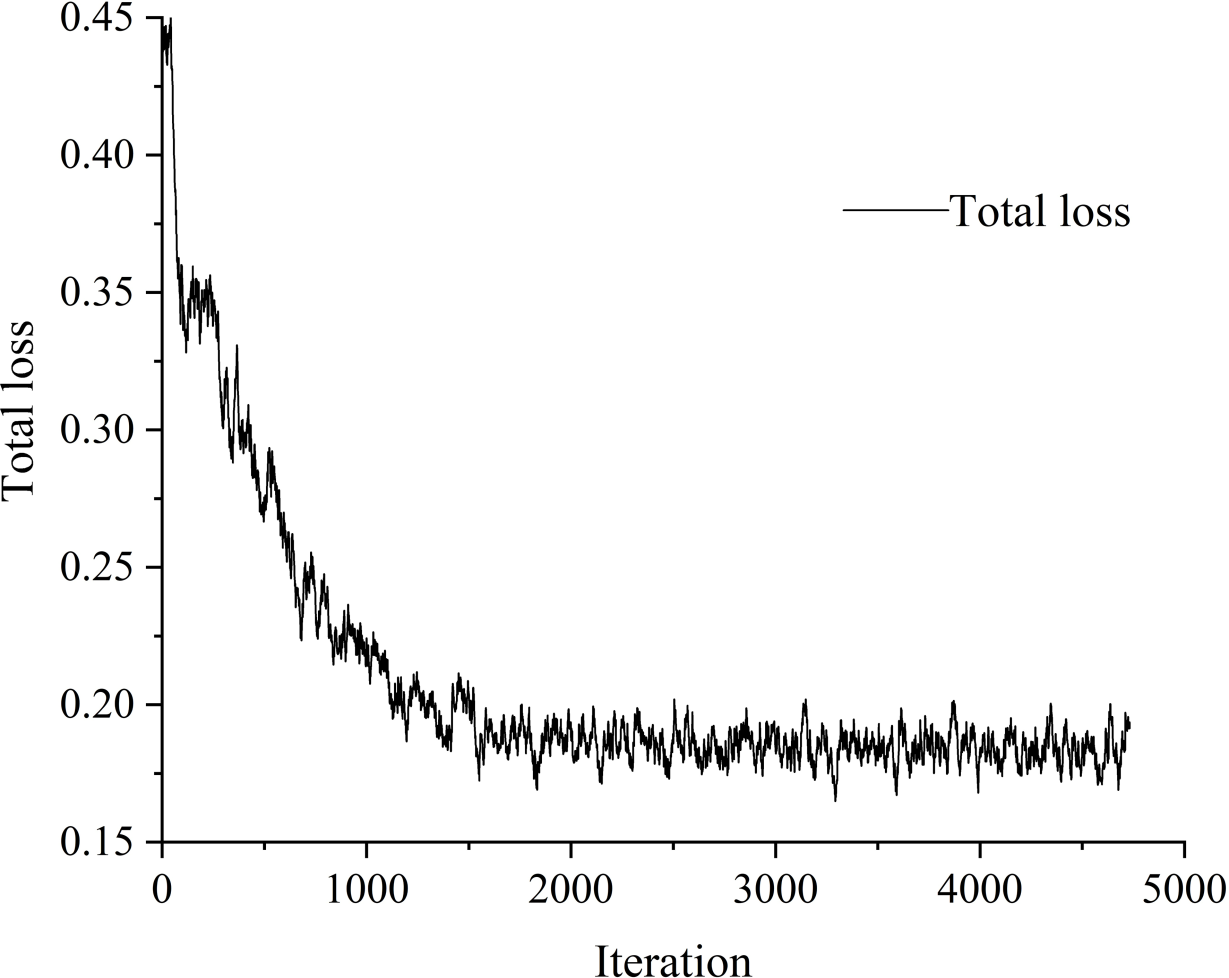
The training process of the tomato leaf detection model.

**Figure 9:**
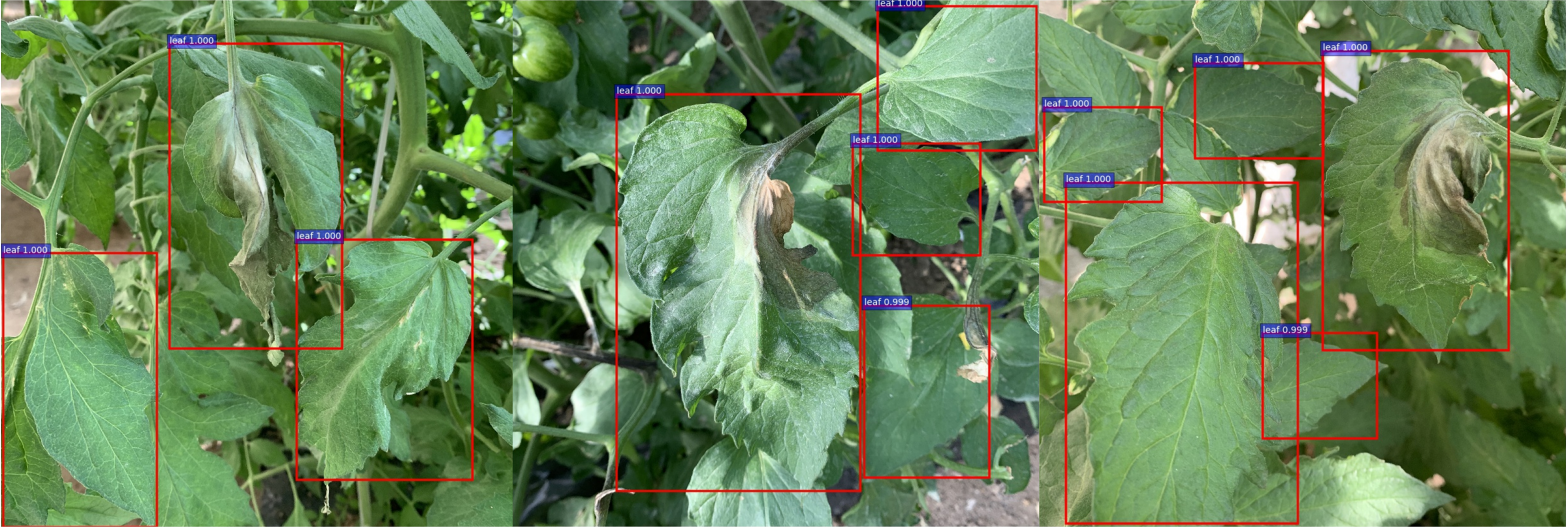
Test results of the tomato leaf detection models in different environments.

The training process and detection results of the tomato leaf detection model are shown in **Figure 8** and **Figure 9**, respectively. During the training process, the total loss function exhibits a downward trend, and the oscillation interval is stable between 0.15-0.20. After the training process is completed, the model can effectively detect the clear leaves in the original collected tomato images while ignoring the blurred leaves and incomplete leaves in the background. The calculated accuracy of the model is 93.7%, the recall is 85%, the weight of the model is 88MB, and which proves that the model has a good detection effect for tomato leaves.

At the same time, to verify the effectiveness of the proposed model, we conducted comparative experiments with YOLO v8x, YOLO v8l, and Faster R-CNN. The accuracy, recall rates, and model sizes are as follows: for the YOLO v8x model, the accuracy is 95%, the recall rate is 85.5%, and the model size is 130 MB; for the YOLO v8l model, the accuracy is 92.5%, the recall rate is 83.2%, and the model size is 84 MB; for the Faster R-CNN model, the accuracy is 94.5%, the recall rate is 85.9%, and the model size is 467 MB. Compared to the original Faster R-CNN, the detection performance of our proposed model is slightly lower, but the model size is only about one-fourth of that. In comparison with YOLO v8x, the model size is approximately two-thirds of that, and there is little difference in detection performance. However, when compared to YOLO v8l, the detection performance is improved by about 2 percentage points, while the model size only increases by 4 MB.

### 4.3 Recognition results for tomato leaf diseases

To verify the effectiveness of the tomato leaf disease recognition model, the original tomato disease recognition training set and the enhanced tomato disease recognition training set are used to train the ultra-lightweight tomato disease recognition model based on the ECA mechanism proposed in this paper. The robustness of the model is tested on the testing set derived from 14 kinds of different environments. To further analyze the impact of the improved module on the recognition results, an ablation experiment is carried out on the ultra-lightweight tomato disease recognition model. Finally, the same parameter configuration and methods are used to train mainstream recognition networks, including the MobileNet series, VGG16, ResNet50 and AlexNet, for a comparison with the model proposed in this article in terms of recognition performance.

#### 4.3.1 Training results of the ultra-lightweight tomato disease recognition model

**Figure 10:**
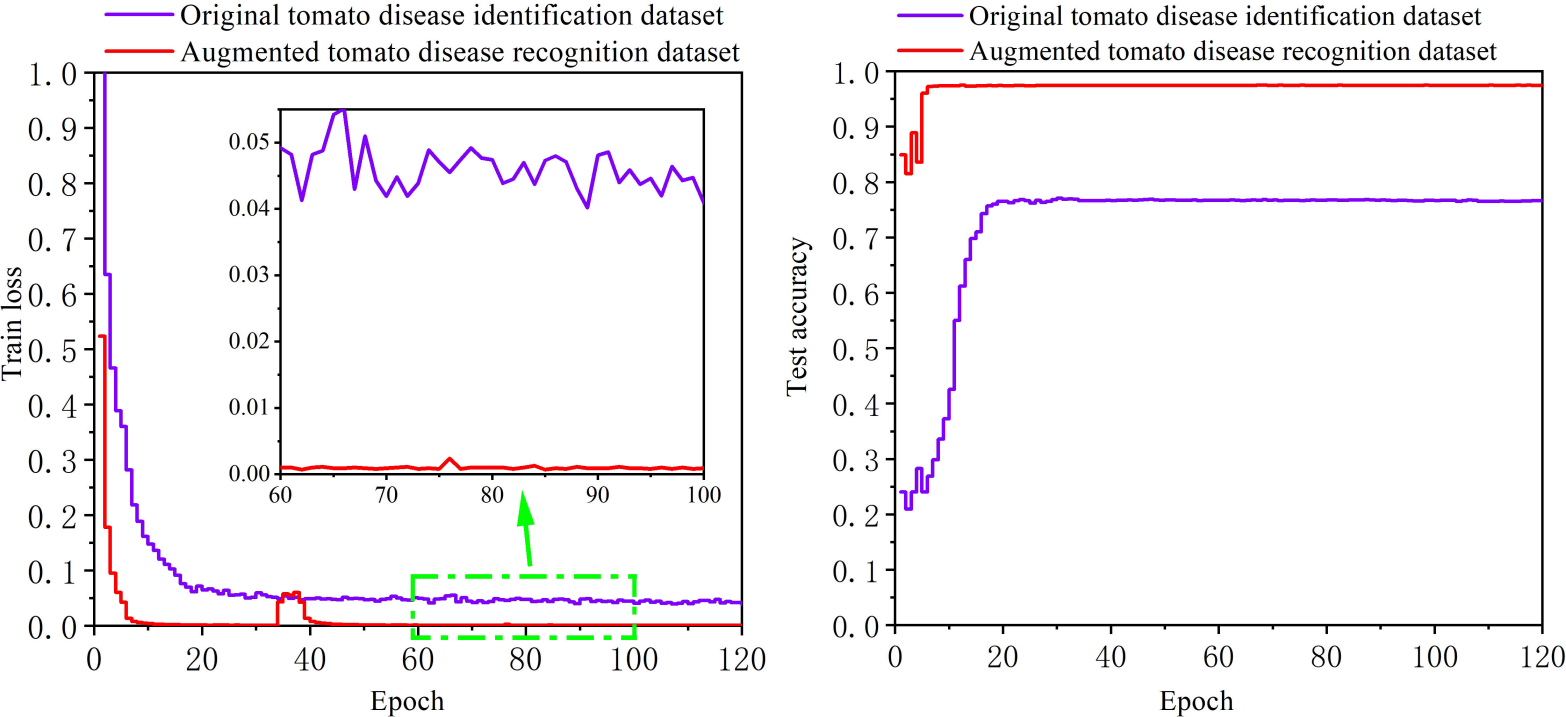
The training results of the ultra-lightweight tomato disease recognition model obtained with different datasets.

The original tomato disease recognition training set and the enhanced tomato disease recognition training set are separately used to train the disease recognition model, and then the trained model is verified on the same enhanced testing set. The image size used for the two training sets is scaled to 448*448 pixels. **Figure 10** shows the change curve of the loss function and the accuracy achieved on the testing set during the training process. As the number of training epochs increases, the loss functions of the model for the two datasets first decrease and then stabilize, and the accuracy on the testing set also increases and then stabilizes. However, the loss function of the model trained on the enhanced tomato disease recognition dataset is lower, and the accuracy on the testing set reaches 97.84%. The accuracy of the model trained on the original tomato disease recognition dataset is only 77.09% on the same testing set, which is far lower than the recognition accuracy of the model trained on the enhanced tomato disease recognition dataset. It is proven that the 13 kinds of image enhancement methods used in this paper can greatly improve the accuracy of the model with respect to the task of tomato disease recognition.

#### 4.3.2 Comparison of the recognition results of each model

**Figure 11:**
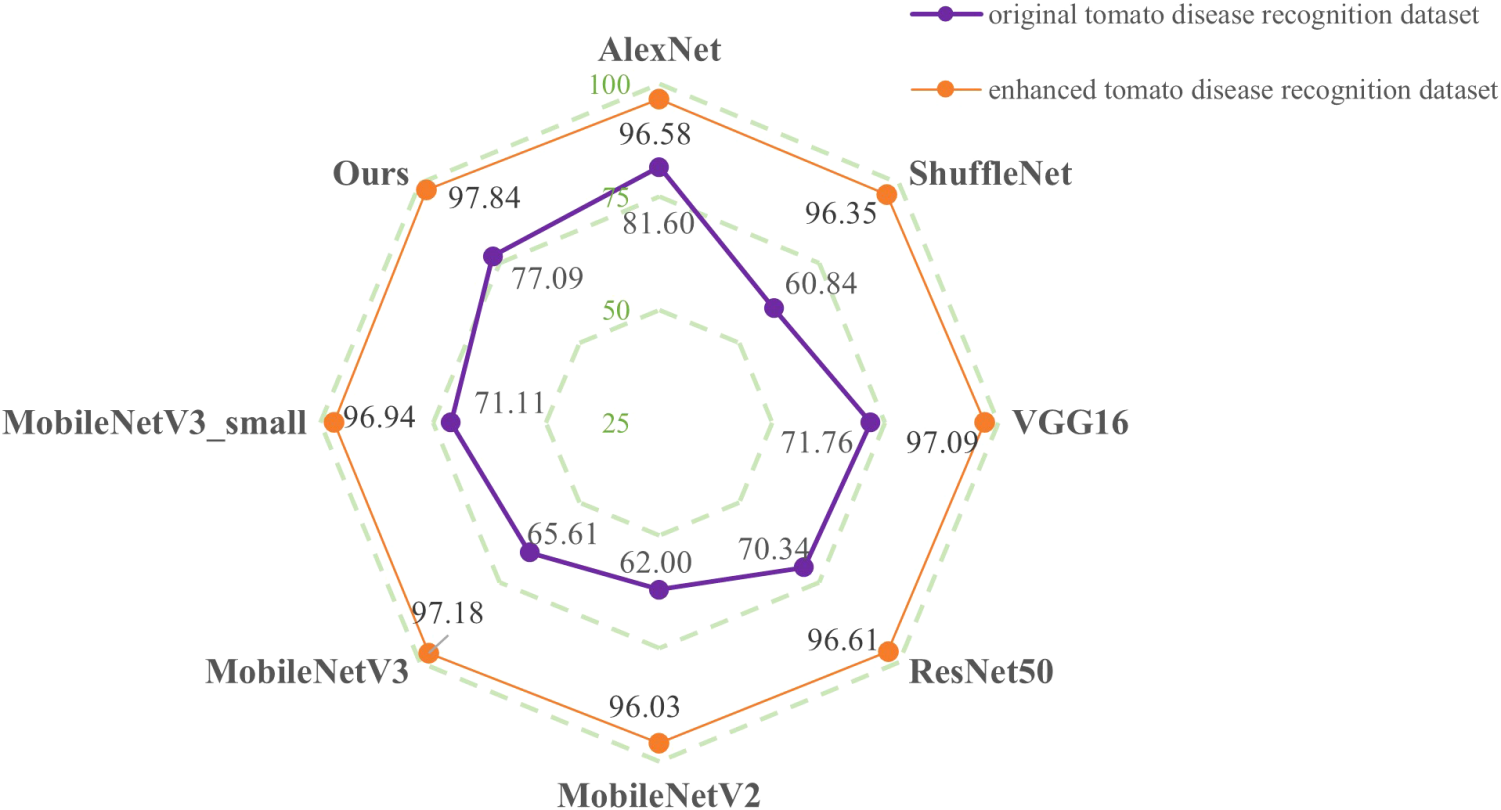
Comparison of the accuracies of different models on the enhanced testing set.

To further verify the influence of the model and enhancement method proposed in this paper on the recognition results, the current mainstream models are trained on the original tomato disease recognition training set and the enhanced tomato disease recognition training set and then uniformly tested on the enhanced testing set. The results are shown in **Figure 11**.

After conducting training on the enhanced tomato disease recognition training set, the recognition accuracies of the tomato disease recognition models in different environments are much higher than those of the models trained on the original tomato disease training set. This is because the enhanced testing set contains images of diseased leaves in a complex environment. Data enhancement can simulate diseased tomato leaves in a complex unconstrained environment, so training models with the enhanced tomato disease dataset can make them more robust. In addition, although the proposed model trained on the original tomato disease dataset has a recognition accuracy of 77.09% on the testing set, which is lower than that of AlexNet, this value is much higher than the test results of other networks. However, the recognition accuracy of the proposed model on the testing set after training on the enhanced tomato disease recognition dataset is 97.84%, which is higher than the recognition accuracies of all other network models. The experimental results prove that the image enhancement method selected in this paper can effectively improve tomato recognition accuracy. When the number of datasets is expanded to 84,014, the shallow network AlexNet exhibits certain limitations and is unable to obtain higher accuracy, and the model proposed in this paper has the highest accuracy among all tested models.

To further study the influence of the utilized complex environment on the models, each type of network is trained on two datasets and then uniformly tested on 14 enhanced tomato disease recognition testing sets. The weight of the version of each model with the highest average accuracy on the testing set is saved, and then the influences of different environments on the accuracy of model recognition are analyzed. The test results obtained after training on the original tomato disease recognition dataset are shown in **Table 3**, and the test results obtained after training on the enhanced tomato disease recognition dataset are shown in **Table 4**.

**Table 3:** Test results obtained after training on the original tomato disease recognition dataset.

	AlexNet	ShuffleNet	VGG16	ResNet50	MobileNet			Ours
					V2	V3 large	V3 small	
Original image	94.58%	90.32%	91.24%	93.83%	85.24%	93.08%	92.16%	95.25%
Vertical flip	94.91%	90.53%	91.49%	93.08%	84.99%	93.66%	92.74%	96.08%
Horizontal flip	88.66%	82.35%	83.32%	87.24%	81.90%	90.16%	89.07%	91.16%
Brightness increase	68.56%	28.73%	50.21%	33.36%	31.61%	29.27%	48.96%	50.71%
Brightness reduction	35.70%	20.56%	50.29%	20.52%	21.52%	21.68%	40.95%	57.38%
Chroma increase	92.91%	73.13%	83.24%	83.32%	75.56%	81.73%	82.74%	87.57%
Chroma reduction	74.65%	40.12%	43.79%	72.39%	43.79%	39.12%	53.88%	58.97%
Contrast increase	91.58%	68.83%	81.32%	69.89%	72.98%	78.73%	79.32%	84.99%
Contrast reduction	79.32%	38.71%	42.95%	31.94%	46.62%	26.77%	29.86%	51.54%
Sharpness increase	94.50%	84.19%	91.08%	93.83%	85.49%	92.91%	92.08%	94.91%
Sharpness reduction	94.50%	84.36%	91.24%	93.91%	85.24%	93.08%	92.16%	95.08%
Laplace transform	94.33%	83.53%	90.16%	93.83%	85.32%	92.91%	91.91%	95.25%
Gamma transform	78.07%	47.39%	78.73%	62.05%	46.54%	54.88%	65.72%	67.31%
CLAHE	52.54%	19.02%	35.53%	55.55%	21.18%	30.61%	43.95%	53.13%
Average accuracy	81.06%	60.84%	71.76%	70.34%	62.00%	65.61%	71.11%	77.09%

**Table 4:** Test results obtained after training on the enhanced tomato disease recognition dataset.

	AlexNet	ShuffleNet	VGG16	ResNet50	MobileNet			Ours
					V2	V3 large	V3 small	
Original image	97.41%	97.08%	97.58%	97.58%	97.00%	98.25%	97.83%	98.58%
Vertical flip	97.00%	97.12%	97.50%	97.08%	97.25%	97.91%	97.83%	98.33%
Horizontal flip	96.25%	95.63%	96.83%	95.91%	95.75%	97.08%	96.25%	97.58%
Brightness increase	94.66%	94.36%	95.16%	93.49%	92.66%	94.75%	94.16%	96.33%
Brightness reduction	97.08%	96.15%	97.16%	95.91%	95.58%	97.08%	96.41%	97.75%
Chroma increase	96.66%	96.02%	97.33%	97.25%	96.00%	97.50%	97.33%	97.50%
Chroma reduction	96.25%	95.94%	97.16%	96.25%	96.16%	96.83%	96.91%	98.17%
Contrast increase	96.58%	96.28%	97.16%	96.91%	96.00%	97.08%	97.08%	97.66%
Contrast reduction	96.91%	96.59%	97.08%	96.66%	96.25%	96.33%	96.33%	97.75%
Sharpness increase	97.41%	97.12%	97.58%	97.66%	96.91%	98.25%	97.75%	98.58%
Sharpness reduction	97.41%	97.16%	97.58%	97.58%	97.00%	98.25%	97.75%	98.58%
Laplace transform	97.25%	96.98%	97.50%	97.66%	97.00%	98.17%	97.66%	98.42%
Gamma transform	96.08%	96.63%	97.58%	96.41%	95.58%	96.66%	96.83%	97.08%
CLAHE	95.16%	95.86%	96.08%	96.16%	95.25%	96.33%	97.08%	97.41%
Average accuracy	96.58%	96.35%	97.09%	96.61%	96.03%	97.18%	96.94%	97.84%

Upon analyzing the recognition results of the models trained on the different sub-datasets of the enhanced tomato disease recognition testing set, it is found that the accuracy of each model on the original images of the testing set is very high. The image flip, sharpness transformation and Laplace transformation have the least influence on the recognition result, because sharpness transformation and Laplace transformation can enhance the details and edge information of the image, while flipping changes the angle of the image and has little effect on its texture and shape. However, changes in the brightness, chroma, and contrast of an image or a gamma or CLAHE transformation reduce the recognition accuracy of the model to varying degrees, mainly because these transformations have a significant impact on the color, texture, and other aspects of the image, and these types of data are missing from the original data. The lack of these types of data in the original data results in the model lacking robustness to this type of noise. Meanwhile, based on the experimental results in **Tables 3**, **4**, we can conclude that the number of model parameters does not necessarily represent the recognition performance of the model. Excessive model parameters may lead to the extraction of redundant features, thereby affecting the performance of the model. On the contrary, a small number of parameters may also reduce the feature extraction capability of the model. It can be seen from **Table 3** that without image enhancement, the recognition accuracy of the model proposed in this paper is higher than that of most models in different environments. Through image enhancement, the model can be adapted to the changes in these tomato images, and the recognition accuracy of the model in different environments can be significantly improved. In **Table 4**, the accuracies of the model proposed in this paper is maintained at approximately 97% in different environments. The recognition accuracy in most environments is higher than that of other models, and the average accuracy is 97.84%, which is higher than that of other models. The experimental results verify that the model proposed in this paper not only has the highest recognition accuracy for tomato disease images in an unconstrained environment but also has better robustness to images with multiple types of noise interference.

The tomato disease recognition model designed in this paper not only has high accuracy and strong robustness in a variety of complex environments but is also ultra-lightweight. **Table 5** compares the number of parameters utilized by each model. The weight of the model proposed in this paper is only 1.6 MB, and the number of parameters is 0.418 M, which is only one-tenth of that required by MobileNetV3. Although the tomato disease recognition accuracy listed in **Table 3** is slightly lower than that of AlexNet, the number of model parameters is reduced by 200 times. After training on the enhanced tomato disease recognition dataset in **Table 4**, the accuracy of the model in this paper ranks first, and the number of parameters is much smaller than that of VGG16 and ResNet50. Compared with the lightweight MobileNet series models proposed in recent years, the model proposed in this paper not only has a higher recognition accuracy but also has a lower model weight. For example, the weight of the model developed in this paper is one-fifth of that of MobileNetV2, but the test results in **Table 3** and **Table 4** show that the recognition accuracies of the model in this paper are 15.09% and 1.44% higher than those of MobileNetV2. Experiments show that the ultra-lightweight tomato disease recognition model based on the ECA module proposed in this paper balances the contradiction between tomato disease recognition accuracy and model complexity and uses the ultra-lightweight network to obtain disease recognition accuracy.

**Table 5:** Model size comparison and evaluation.

	AlexNet	ShuffleNet	VGG16	ResNet50	MobileNet			Ours
					V2	V3 large	V3 small	
Model weight	217MB	7.6MB	268MB	89.9MB	8.69MB	16.2MB	2.32MB	1.68MB
Parameter	57M	1.98M	70M	23M	2.23M	4.2M	0.588M	0.418M
Test results in **Table 3**	81.06%	60.84%	71.76%	70.34%	62.00%	65.61%	71.11%	77.09%
Test results in **Table 4**	96.58%	96.35%	97.09%	96.61%	96.03%	97.18%	96.94%	97.84%

#### 4.3.3 Results of an ablation experiment

In this paper, an ablation experiment is designed to further explore the effect of the high-efficiency channel attention mechanism and dropout layer on the obtained tomato disease recognition accuracy. After the ECA mechanisms in all modules are removed, the formed tomato disease recognition model is called No_ECA. After the last dropout layer in the model proposed in this paper is removed, the resulting tomato disease recognition model is called No_Dropout. These two models are trained on the enhanced tomato disease recognition dataset, and the models are tested according to the test method described in section 4.3.2. The test results are shown in **Table 6**.

**Table 6:** Comparison of the ablation experiment results for the model proposed in this paper.

	Original image	Vertical flip	Horizontal flip	Brightness increase	Brightness reduction	Chroma increase	Chroma reduction	Contrast increase
No_ECA	97.50%	97.66%	96.16%	94.91%	96.75%	97.33%	97.16%	97.41%
No_Dropout	97.83%	97.50%	97.25%	95.50%	97.16%	97.91%	97.83%	97.00%
Ours	98.58%	98.33%	97.58%	96.33%	97.75%	97.50%	98.17%	97.66%
	Contrast reduction	Sharpness increase	Sharpness reduction	Laplace Transform	Gamma transform	CLAHE	Average accuracy	Parameter
No_ECA	97.00%	97.58%	97.50%	97.66%	96.75%	96.41%	96.99%	418148
No Dropout	97.58%	97.91%	97.83%	97.91%	97.25%	97.16%	97.40%	418181
Ours	97.75%	98.58%	98.58%	98.42%	97.08%	97.41%	97.84%	418181

According to the results of the ablation experiment, when using the same dataset and training configuration to train No_ECA and No_Dropout, their recognition accuracies are lower than that of the model proposed in this paper. Although a large number of ECA modules are used in the model proposed in this paper to improve the accuracy of tomato disease recognition, the number of model parameters increases by 33, which is only a slight increase. This proves that our model not only improves the accuracy of tomato disease recognition but also realizes ultralightweight characteristics. It balances the contradiction between model accuracy and complexity and has strong robustness to complex and unconstrained environments.

## 5 Discussion and conclusion

This paper addresses the limitations of existing tomato disease recognition models, which are typically capable of identifying only a single diseased leaf and struggle to reconcile the conflicting demands of accuracy and complexity. To overcome these challenges, we propose a comprehensive framework that integrates tomato leaf detection with leaf disease recognition. Initially, we develop a tomato leaf detection model designed to extract individual diseased leaves from tomato disease images captured in real-world, unconstrained environments. Subsequently, we introduce an effective data enhancement method that simulates image noise across various settings, thereby augmenting the model’s robustness in complex environments. Finally, we present an ultra-lightweight tomato leaf disease recognition model tailored for operation in such challenging conditions. This model leverages a high-efficiency channel attention mechanism and incorporates five inverted residual modules to enhance accuracy while minimizing the number of parameters, effectively balancing model complexity with recognition accuracy and reducing hardware resource consumption. We utilize both the original tomato disease dataset and an enhanced version to train the model, subsequently evaluating its recognition accuracy across 14 distinct noise environments. Experimental results reveal that our ultra-lightweight tomato disease recognition model, based on high-efficiency channel attention, achieves an accuracy of 97.84% while maintaining only 0.418 million parameters. In comparison to traditional image recognition models such as AlexNet, VGG16, ResNet50, and the MobileNet series, the proposed model not only demonstrates superior accuracy across the 14 noisy environments but also significantly reduces the parameter count. Additionally, our model addresses the limitation of existing systems that can only recognize a single disease image, making it suitable for deployment on mobile devices for practical demonstration applications. At the same time, based on the framework proposed in this study, using disease images of other crops for model fine-tuning can also be applied to other crops. Although the existing models have achieved good results, there is still room for improvement in future research. Firstly, the existing image data mainly simulates complex environments through different data processing methods. In the future, more image acquisition devices can be used to obtain image data under different performance devices, so that the images are closer to the natural environment. At the same time, more complex leaf disease images can be collected to establish a more balanced leaf disease dataset. Secondly, based on this model, techniques such as compression pruning, knowledge distillation, and quantification can be used to further improve the lightweight process of the model.

## Data Availability

The raw data supporting the conclusions of this article will be made available by the authors, without undue reservation.
